# Effects of cyclooxygenase-2 gene silencing on the biological behavior of SKOV3 ovarian cancer cells

**DOI:** 10.3892/mmr.2021.12346

**Published:** 2021-08-08

**Authors:** Feng-Jun Guo, Jing-Yan Tian, Yue-Mei Jin, Ling Wang, Rui-Qi Yang, Man-Hua Cui

Mol Med Rep 11: 59-66, 2015; DOI: 10.3892/mmr.2014.2732

Following the publication of the above article, the authors have requested that it be retracted. They alerted the Editorial Office to the fact that the same data, albeit with a different view, had been selected to show the ‘CON’ and ‘NC’ experiments for the colony-formation assays featured in Fig. 6. The Editor has agreed to the authors’ request that the paper be retracted. All the authors agree to this retraction, and apologize for any inconvenience caused.

